# How We Fail Children With Developmental Language Disorder

**DOI:** 10.1044/2020_LSHSS-20-00003

**Published:** 2020-08-05

**Authors:** Karla K. McGregor

**Affiliations:** a Center for Childhood Deafness, Language and Learning, Boys Town National Research Hospital, Omaha, NE; b Department of Communication Sciences and Disorders, The University of Iowa, Iowa City

## Abstract

**Purpose:**

For over two decades, we have known that children with developmental language disorder (DLD) are underserved. We have also known that DLD does not attract the research attention that it merits given its prevalence and impact. The purposes of this clinical focus article are to present evidence that these failures continue, explore the reasons behind these failures, and propose solutions.

**Method:**

I reviewed the literature and applied bibliometric analysis procedures from Bishop (2010) to quantify research efforts aimed at DLD compared to other neurodevelopmental disorders.

**Results:**

The percentage of children who are deemed eligible for clinical services because of DLD continues to fall well short of estimates based on the prevalence of DLD in community samples. The amount of research conducted on DLD relative to other neurodevelopmental disorders remains low. Contributing factors include a lack of awareness of DLD, the hidden nature of DLD, entrenched policies, and the dissonance created when speech-language pathologists must diagnose DLD in school settings.

**Conclusions:**

Expanded approaches to supporting children with DLD are required. These might include engagement in advocacy and awareness campaigns; clearer communication with the families we serve and enhanced collaborations with classroom teachers; the implementation of school-based language screenings; participation in policymaking; and the development of service delivery models that operate alongside those that exist in our schools and complement their function.

**Supplemental Material:**

https://doi.org/10.23641/asha.12743273

Developmental language disorder (DLD) is a neurodevelopmental condition that emerges in early childhood and frequently persists into adulthood. People with DLD have significant difficulty learning, understanding, and using spoken language. Under U.S. Public Law 101-476 (Individuals with Disabilities Education Act [IDEA], 2004; first issued in 1990 and reissued in 2004), children may be eligible for school-based services, typically under the category “speech-language impairment,” if their DLD affects educational performance and requires specially designed support. DLD is one of the most common neurodevelopmental disorders. With an estimated prevalence of 7.58% (Norbury et al., 2016; see also Tomblin et al., 1997), it is nearly 7 times more common than autism spectrum disorder (ASD; prevalence = 1.1%; Brugha et al., 2012) and 46 times more common than permanent childhood hearing impairment (prevalence = 0.165%; Fortnum et al., 2001).

As a population, people with DLD face significant risks. Compared to other students, those with DLD are 6 times more likely to have reading disabilities, 6 times more likely to have significant spelling problems, 4 times more likely to struggle with math, and 12 times more likely to face all three of these difficulties combined (Young et al., 2002). People who have DLD are 6 times more likely than others to experience clinical levels of anxiety and 3 times more likely to have clinical depression (Conti-Ramsden & Botting, 2008). Girls with DLD are 3 times more likely to experience sexual abuse (Brownlie et al., 2007). Boys with DLD are 4 times more likely to engage in delinquent behavior (Brownlie et al., 2004). Adults with DLD are twice more likely to go over a year without employment than other adults (Law et al., 2009).

Without a doubt, DLD is a common condition that limits the health, happiness, and success of many who live with it. Nevertheless, people with DLD are underserved, and the condition itself is under-researched. The reasons are complicated, but the consequences of continued failure are dire. This clinical focus article is a call to action. I will provide evidence to demonstrate the ways that we, as a profession, are failing children with DLD; explore the reasons for these failures; and encourage change. The institutions and policies that dictate, support, or constrain clinical services and research efforts vary widely from country to country. This review is admittedly United States–centric, with some attention paid to the United Kingdom as well, but it is my hope that some of the points raised here are universally relevant.

## Children With DLD Are Underserved

In 1997, Tomblin et al. identified 216 kindergarten children within a community sample who scored more than 1.25 *SD*s below the mean on two or more of five composite scores that captured receptive and expressive language abilities in the lexical, grammatical, and narrative domains. Only a minority of those children had ever been flagged with language concerns.

Of course, cutoffs on norm-referenced tests vary across settings, and functional impact as ascertained from a variety of sources such as language samples, achievement tests, response to intervention (RTI) or dynamic assessments, parent reports, and teacher observations must, by law, also be considered part of eligibility determination in U.S. schools (Ireland et al., 2013). Therefore, it is likely that not each of these low-scoring children should have raised concerns. In fact, some of them did not continue to present with low scores in subsequent years. Let us be conservative. If we take only the poorest performers—those who scored below the 3rd percentile, a low level of performance indeed—only 39% of them had ever been identified as having language deficits (Tomblin et al., 1997). Given the high risks of academic and social failure associated with DLD, it is astounding that a majority of likely affected children were not identified and, therefore, were not receiving services.

Unfortunately, the situation was no better 19 years later when Norbury et al. (2016) collected a community sample of first graders in England. Of the children they identified as meeting the criteria for DLD, only 3.5% had a Statement of Special Educational Needs (the U.K. equivalent of an Individualized Education Program in the United States), and only 39% were receiving language intervention outside of school (Norbury et al., 2016). Although we were alerted to the situation more than 20 years ago, we continue to underserve children with DLD.

Worse still, some are more likely to go without service than others. Morgan et al. (2017) analyzed data from two nationally representative cohorts of U.S. kindergartners, one collected in 1998–1999 (*N* = 16,800) and another collected in 2010–2011 (*N* = 12,080), to determine whether receipt of special education services for children deemed eligible under the category of speech-language impairment varied with family and child characteristics, academic achievement, and behavioral health. Numerous, long-standing inequities were evident.

### Inequities Related to Gender

Morgan et al. (2017) found that boys were significantly more likely to receive services than girls in 1999 and 2011. This disproportionality also characterizes language services in U.K. schools (Lindsay & Strand, 2016). To some extent, the larger representation of boys could be fair given that community samples do reveal a higher prevalence of DLD among boys than girls; however, the difference is small and not always significant (Norbury et al., 2016). The estimated male-to-female prevalence ratio within the DLD population is 1.3:1 (Tomblin et al., 1997), whereas the male-to-female receipt-of-services ratio is 1.71:1 in the United States (Morgan et al., 2017) and 2.55:1 in the United Kingdom (Lindsay & Strand, 2016). Girls and boys may present with different profiles of strengths and weaknesses in language itself (McGregor et al., 2020) or differences in social behavior that serve to magnify (in the case of boys) or hide (in the case of girls) their language weaknesses (Hart et al., 2004; Toseeb et al., 2017).

### Inequities Related to Behavioral Profile

In 1999 and again in 2011, children who had poor self-regulation were more likely to receive speech-language services than those who had better self-regulation (Morgan et al., 2017). This discrepancy may reflect the extent of comorbidities between DLD and emotional–behavioral disorders (Benner et al., 2009). However, it might also indicate that children with DLD who behave well in the classroom escape notice, a likely situation given that community samples reveal lower rates of behavior problems among children with DLD than clinical samples (Plomin et al., 2002).

### Inequities Related to Geographic Location

At both time points, geographic inequities were evident in that children from the Western United States were less likely to receive services than children from the Northeastern United States (Morgan et al., 2017). From 2004 to 2016, 60%–90% of school speech-language pathologists (SLPs) from the Mountain and Pacific states reported that job openings exceeded job seekers, indicating widespread, long-standing shortages of speech-language services in the Western United States (American Speech-Language-Hearing Association [ASHA], 2016a, 2016b). In a 2006 survey of school SLPs (*N* = 1,644), 79% reported that shortages resulted in increased caseload size, 56% reported decreased quality of service, and 55% reported decreased opportunities for individual (one-on-one) service delivery (ASHA, 2006).

### Inequities Related to Minority Status

Other disparities reported in Morgan et al. (2017) involved majority–minority linguistic, racial, and ethnic divisions. At both time points, children who spoke English as an additional language were about 50% less likely to be identified as service eligible than monolingual English speakers. In the United States, most SLPs are monolingual, and, as a profession, we lack tools that are appropriately normed for people who speak English as an additional language. Both are likely barriers to identification. Although Hispanic children were not significantly underserved in 1999, this had changed by 2011 when their odds of receiving services were 46% lower than the odds for non-Hispanic children (Morgan et al., 2017). In 1999, the odds that a Black child would receive services were 61% lower than the odds for a White child. This disparity remained in 2011 (Morgan et al., 2017). An examination of data collected from 2009 to 2014 revealed continued disproportionality, with 62% of states underrepresenting Black students and 14% of states overrepresenting them (Robinson & Norton, 2019). Note that these linguistic, racial, and ethnic disparities are *not* a matter of the socioeconomic disadvantage that, unfortunately, is more common among minority populations in the United States (although this plays a role as well; see the Inequities Related to Socioeconomic Status section). Morgan et al. took pains to adjust each estimator variable—in this case, using a minority language, being Hispanic, and being Black—for confounding factors that could muddy the interpretation of the data, that is, factors that included household income, parents' education levels, and even the child's own academic and behavioral performance.

### Inequities Related to Socioeconomic Status

Socioeconomic status (SES) was not a significant predictor of receipt of services in the work of Morgan et al. (2017), but it does emerge as predictive in other studies. Wittke and Spaulding (2018) compared two groups of preschoolers. Preschoolers in both groups had DLD, but despite similar severity of presentation, one group received intervention, and the other did not. Two characteristics set them apart. One was that the children receiving intervention were judged as having poorer executive control by their teachers, this being akin to the self-regulation variable reported in the work of Morgan et al. The other was that the children in receipt of services had mothers who are better educated, and this was a large effect. Bishop and McDonald (2009) also compared children whose scores on standardized tests were indicative of DLD but who varied in receipt of services. In this sample as well, the SES of their mothers differentiated the children, with higher SES being characteristic of children who received services. Receipt of services was not related to the severity of the language problem itself (in fact, on a test of reading comprehension, those who received no services scored lower than those who did) or to the children's nonverbal IQ performance. There could be many issues at play here, but the obvious one is the extent to which these mothers had access to the resources that would allow them to seek help for their children. Although U.S. law mandates free school-based services for children with disabilities, navigating the process of requesting and determining the right course of care is not free. It can be costly in terms of time spent away from work and transportation expenses to school meetings as well as complicated in terms of the legal language, educational jargon, and health literacy issues.

Inequities related to SES extend beyond the school–house doors. Some families can, and do, supplement school-based services with treatments in outpatient, private practice, or university training clinics. However, these families are exceptions because health insurance rarely pays for these labor-intensive; long-term; and, thus, costly services. Goodwin (2016) surveyed parents whose children were receiving speech-language services in private clinics. All of the families had health insurance. Nevertheless, parents reported that they faced numerous barriers when securing services for their children, including cost, lack of knowledge of available resources, and lack of time. Parents with lower SES faced more barriers than parents with higher SES, and, not surprisingly given this situation, the children from lower SES families accessed private services later than the children from higher SES families (Goodwin, 2016).

## DLD Is Under-Researched

In 2010, Bishop authored a paper titled “Which neurodevelopmental disorders get researched and why?” She searched Web of Science, an extensive repository of published research, for titles that included the names of 35 neurodevelopmental disorders. A primary question concerned the attention given to DLD (at that time, usually called specific language impairment) relative to the other disorders. To make a fair comparison, she considered two variables: prevalence, given the logic that more prevalent disorders should be studied more often than those less prevalent, and severity, given the logic that more severely disabling disorders should be studied more often than those less severe. Fortunately, the two are inversely related; severely disabling conditions are rare.

**Figure 1.**
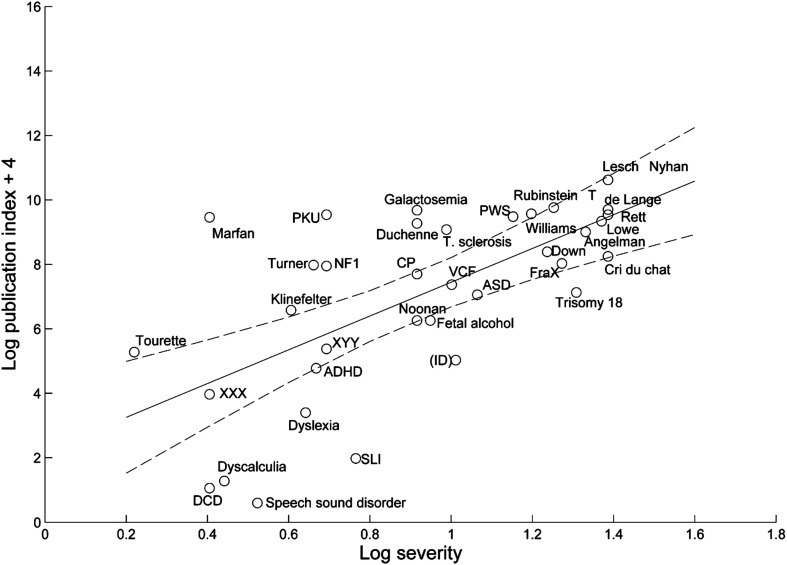
Regression of log publication index on log severity for the years 2000–2009, with a 95% confidence interval shown with dotted lines, reproduced from Bishop (2010). A constant of 4 is added to the log publication index to avoid negative numbers. ADHD = attention-deficit/hyperactivity disorder; ASD = autism spectrum disorder; CP = cerebral palsy; DCD = developmental coordination disorder; de Lange = Cornelia de Lange syndrome; FraX = fragile X syndrome; ID = intellectual disability (shown in parentheses to indicate that the publication index is overestimated); NF1 = neurofibromatosis type 1; PKU = phenylketonuria; Rubinstein T = Rubinstein–Taybi syndrome; SLI = specific language impairment; T. sclerosis = tuberous sclerosis; VCF = velocardiofacial syndrome. Figure reprinted from Bishop (2010) via Creative Commons Attribution license CC BY 4.0.

As predicted, severity was a positive predictor of publication rate (see Figure 1), and the consideration of prevalence estimates further strengthened that prediction. DLD, as well as dyslexia, dyscalculia, developmental coordination disorder, and speech sound disorder, received less research attention than merited given their prevalence and severity of impact. ASD and attention-deficit/hyperactivity disorder (ADHD) receive “appropriate” attention, and single-gene-based disorders tended to receive more attention than expected. To make these discrepancies meaningful, Bishop (2010) derived a publication index for each condition by considering the number of publications per the estimated number of cases in the United Kingdom. For example, from 1985 to 2009, for every 100 individuals in the United Kingdom affected, there were 254.41 papers published on phenylketonuria; 234.42, on Marfan syndrome; 21.39, on ASD; and 2.19, on ADHD. During that same period, there were 0.13 papers per 100 individuals affected by DLD.

To determine whether we have made progress in the years since the work of Bishop (2010), I repeated her procedures using the same publication repository, search terms, prevalence and severity estimates, and weighting procedure. The search terms and database specifications appear in Supplemental Material S1. To compare equal time units, I ran the analysis for the years 2000–2009, that is, the last 10 years covered by Bishop (2010), and for the 10 years since then, that is, 2010–2019. Note that, because the population of the United States is larger than that of the United Kingdom, the publication index results for the United States cannot be directly compared with those in the work of Bishop (2010). Instead, the critical question of interest is whether there were improvements in the research attention paid to DLD between the decades 2000–2009 and 2010–2019 when both have a common denominator, that is, the estimated number of cases in the U.S. population. The literature itself reflects work worldwide, so although the exact numbers are specific to the United States, the relative change over time is universally relevant.

**Figure 2.**
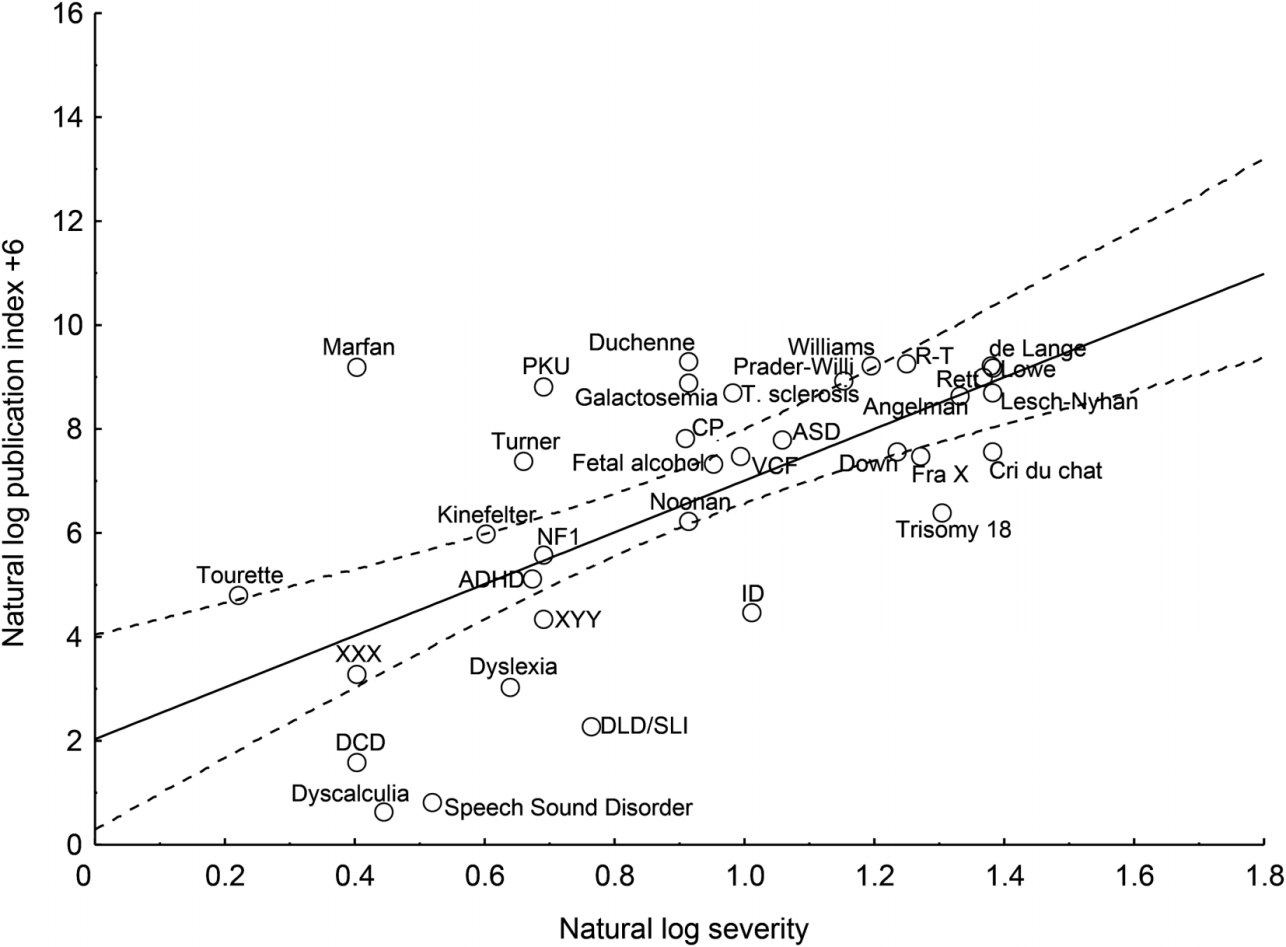
Regression of log publication index on log severity for the years 2010–2019, with a 95% confidence interval shown with dotted lines. A constant of 6 is added to the log publication index to avoid negative numbers. ADHD = attention-deficit/hyperactivity disorder; ASD = autism spectrum disorder; CP = cerebral palsy; DCD = developmental coordination disorder; de Lange = Cornelia de Lange syndrome; DLD/SLI = developmental language disorder/specific language impairment; FraX = fragile X syndrome; ID = intellectual disability; NF1 = neurofibromatosis type 1; PKU = phenylketonuria; R-T = Rubinstein–Taybi syndrome; T. sclerosis = tuberous sclerosis; VCF = velocardiofacial syndrome.

As in the work of Bishop (2010), the publication index for 2010–2019 was predicted by severity, *R* = .65, *p* < .0003 (see Figure 2). Adding prevalence to the regression model accounted for further variance in the publication index, *R* = .85, *p* < .0001.

The number of publications in the two decades under consideration, the percentage of change in the number of publications from one decade to the next, the estimated number of cases in the United States, and the publication index appear in Table 1. With the single exception of Lesch–Nyhan syndrome, all neurodevelopmental conditions have received more research attention in the most recent decade than in the decade that preceded it. The number of publications about Duchenne muscular dystrophy, fragile X syndrome, cerebral palsy, and developmental coordination disorder more than doubled. The number of publications about ASD tripled. The percentage increase in publications averaged over all neurodevelopmental disorders was 68% (*SD* = 48). There was a 61% increase in the number of papers devoted to DLD. Thus, the standing of DLD relative to all other neurodevelopmental conditions has changed little since Bishop's (2010) analysis. From 2010 to 2019, there were 0.03 papers on DLD published for every 100 affected children in the United States (see Table 1, “publication index” column). To enable a comparison to the data in the work of Bishop (2010), note that, in terms of the U.K. population, there were 0.16 publications for every 100 affected children (number of children in the United Kingdom = 11,970,367; GOV.UK, 2018) during the years 2010–2019.

**Table 1.** Prevalence, severity rating, number of publications (2000–2009), number of publications (2010–2019), percentage of change from one decade to the next, estimated number of affected children in the United States, and publication index for each condition, ordered by prevalence.

Disorder	Prevalence per 100^a^	Mean severity^a^	No. of pubs (2000–2009)	No. of pubs (2010–2019)	% Change^b^	No. of cases in U.S. (2019)^c^	Pub index^d^
Lesch–Nyhan syndrome	0.0005	4	76	73	−3.95	369	19.78
Lowe syndrome	0.0005	4	72	111	54.17	369	30.08
Rubinstein–Taybi syndrome	0.0008	3.5	116	203	75.00	590	34.38
Cornelia de Lange syndrome	0.0014	4	220	323	46.82	1,033	31.26
Cri du chat syndrome	0.002	4	60	89	48.33	1,476	6.03
Galactosemia	0.002	2.5	305	336	10.16	1,476	22.76
Angelman syndrome	0.004	3.79	349	543	55.59	2,952	18.39
Williams syndrome	0.0044	3.31	787	1,125	42.95	3,247	34.65
Marfan syndrome	0.0067	1.5	930	1,598	71.83	4,945	32.32
Prader–Willi syndrome	0.0067	3.17	970	1,209	24.64	4,945	24.45
Rett syndrome	0.008	3.94	946	1,524	61.10	5,904	25.81
PKU	0.01	2	1,276	1,641	28.61	7,380	22.24
Duchenne muscular dystrophy	0.0143	2.5	1,436	3,929	173.61	10,553	37.23
Tuberous sclerosis	0.0167	2.69	1,432	2,464	72.07	12,325	19.99
Trisomy 18	0.025	3.7	251	347	38.25	18,450	1.88
Velocardiofacial syndrome	0.025	2.72	589	1,062	80.31	18,450	5.76
Turner syndrome	0.04	1.94	1,017	1,548	52.21	29,520	5.24
XYY	0.0545	2	86	101	17.44	40,221	0.25
XXX	0.055	1.5	31	35	12.90	40,590	0.09
Noonan syndrome	0.0571	2.5	399	687	72.18	42,140	1.63
Fragile X syndrome	0.0615	3.57	937	2,554	172.57	45,387	5.63
Kinefelter syndrome	0.086	1.83	430	805	87.21	63,468	1.27
Fetal alcohol syndrome	0.1	2.58	576	748	29.86	73,800	1.01
Cerebral palsy	0.15	2.5	4,367	9,226	111.27	110,700	8.33
Down syndrome	0.1667	3.44	5,224	7,677	46.96	123,025	6.24
Neurofibromatosis type 1	0.308	2	1,028	1,994	93.97	227,304	0.88
Tourette syndrome	0.5	1.25	952	1,480	55.46	369,000	0.40
Autism spectrum disorder	0.65	2.9	12,267	38,110	210.67	479,700	7.94
Developmental dyscalculia	3	1.56	81	137	69.14	2,214,000	0.01
ADHD	5	1.95	10,686	19,992	87.09	3,690,000	0.54
Intellectual disability	5.5	2.75	7,792	11,338	45.51	4,059,000	0.28
Developmental dyslexia	6	1.9	2,151	3,047	41.66	4,428,000	0.07
Developmental coordination disorder	6.5	1.5	291	764	162.54	4,797,000	0.02
Developmental language disorder	7.4	2.15	861	1,388	61.21	5,461,200	0.03
Speech sound disorder	10	1.69	280	523	86.79	7,380,000	0.01

*Note.* PKU = phenylketonuria; ADHD = attention-deficit/hyperactivity disorder.

a As reported in Bishop (2010).

b Formula: [(#2010to19 pubs − #2000to09 pubs)/#2000to09 pubs] × 100.

c Formula: (73,800,000 U.S. children × prevalence per 100)/100; source: https://www.childstats.gov/americaschildren/tables/pop1.asp.

d Number of publications in the decade 2010–2019 per 100 affected cases in the United States based on the population of 73.8 million: (#pubs)/(#cases/100).

How is it that we are failing to serve the majority of children who have DLD? Why does DLD receive relatively little attention from the research community? The reasons are complex, systemic, and intertwined. I will explore four and offer some solutions.

## Reasons

### Reason 1: DLD Is an Unknown Disorder

Despite its high prevalence and significant impact, DLD is a relatively unknown problem. Consider an educated layperson—your neighbor or cousin or your dentist or accountant. Have all of them heard of autism, ADHD, or dyslexia? Have any of them heard of DLD?

In the research literature, children with clinically significant language concerns are often referred to as having a language delay if they are preschoolers, a developmental period when a diagnosis of DLD may well be premature. School children are most often described as having DLD or specific language impairment in the literature. That said, Bishop (2014) found 32 different terms used for DLD in the research literature. Within the research community, debates continue about the most accurate term to use for the condition (Bishop, 2017).

Terms vary among clinicians as well. The *Diagnostic and Statistical Manual of Mental Disorders* (5th ed.; American Psychiatric Association, 2013) labels the problem “expressive–receptive language disorder” or “expressive language disorder,” depending on presentation. In U.S. billing codes, these categories also apply. In U.S. schools, younger children with language concerns likely qualify for services under the category of “developmental delay,” whereas older children will qualify under the category of “speech-language impairment” or, in some cases, “specific learning disability.” In U.K. schools, the educational category is termed “speech, language and communication needs.” To further complicate matters, neurodevelopmental disorders do not fall into mutually exclusive categories. Comorbidities are common; for example, a child might have DLD and ADHD (Tomblin & Mueller, 2012). Moreover, the primary diagnosis might shift over development; the problem best labeled as a language delay at 3 years might be better termed as DLD at 6 years and as specific learning disability at 15 years. The cacophony of terms and the extent to which they change with setting and time impose barriers on our awareness of DLD.

#### A Solution

It is essential to help parents understand the diversity of labels that may apply to their child in various settings and at various times. Without this understanding, parents will find it challenging to communicate with the different professionals who serve their children, and they will be unable to find other families who share their experience.

Moreover, we must guide parents to evidence-based information about language development and disorder so that they can understand their child's needs. Informational resources for parents are growing in availability. These include DLDandMe (https://www.dldandme.org/); Afasic (https://www.afasic.org.uk/); RADLD (https://radld.org/resources/); and, for a broader focus, Understood (https://www.understood.org/en). DLDandMe is aimed at a North American audience; Afasic, at a U.K. audience; and RADLD, at an international audience. RADLD offers a subset of materials in numerous languages. Understood deals mostly with policies pertaining to children with learning disabilities, ADHD, and related conditions in the United States. They offer materials in English and Spanish.

An additional recommendation is to emphasize that DLD, no matter what term is used to refer to it, is a problem of *language,* spoken or written. Persons who are not trained in language science—which typically include parents, teachers, pediatricians, and policy makers—often confuse language with speech or erroneously conclude that language problems are synonymous with intellectual disability. Explaining what language is, which language behaviors are cause for concern, and why language matters will reduce confusion and increase understanding. The term *language* is also broad enough to capture the various ways that DLD manifests over developmental time and from individual to individual, thus helping laypeople understand the connection between seemingly disparate phenomena such as being late to talk and having difficulty learning to spell.

### Reason 2: DLD Is a Hidden Impairment

Zhang and Tomblin (2000) compared the receipt of services for children who had speech sound disorder, DLD, or DLD + speech sound disorder. The children with speech sound disorder, alone or in combination with DLD, were more likely to receive services than children with DLD alone. Specifically, the odds of a child with a speech sound disorder being identified and served were 2.4 times greater than the odds of a child with DLD. The same disparity characterized an Australian sample of children with speech or language disorders (Skeat et al., 2010).

Are children with speech problems more often identified because their problem is more impactful than DLD? No. Compared to speech problems, language problems are stronger predictors of difficulties with reading comprehension and social function in school, at home, and in the community (Zhang & Tomblin, 2000). Young et al. (2002) followed 5-year-olds with speech sound disorder or DLD into early adulthood. They found that those with speech sound disorder were virtually indistinguishable from typical children in their academic outcomes, the one exception being lower word identification during reading. In comparison, children with DLD went on to falter in all academic areas measured—mathematical calculation, spelling, reading comprehension, word identification, and word attack—even after controlling for IQ. This finding coincides with a 30-year follow-up of Danish children with speech-language impairments. In that study, the least useful of the 10 verbal and nonverbal predictors of adult educational and professional outcomes was the children's expressive phonology (Elbro et al., 2011).

We are doing a better job at identifying children with speech sound disorder because the problem is apparent to our ears, as well as the ears of their parents and teachers (Burroughs & Tomblin, 1990), and not because these children are at higher academic or social risk than children with DLD. For the most part, children with DLD do not sound unusual; they may seem younger than they are, but not unusual. By the time they reach school, most can carry on a basic conversation, follow a simple command, and answer a routine question.

Consequently, DLD often remains hidden from the two groups of adults who are a child's primary advocates when it comes to securing services: parents and teachers. Although parents are experts about their children, they are not reliable at judging whether their elementary-aged child's language development is on track relative to that of other children (Hendricks et al., 2019), and in many cases, neither are teachers. Antoniazzi et al. (2010) asked 15 primary school teachers to rate the language and communication abilities of their first-grade students (*N* = 149) via a checklist. When compared to the results of a language screening test, the teachers' ratings were low in both sensitivity and specificity. Teachers themselves report that they are ill-prepared to identify spoken language problems (Dockrell & Lindsay, 2001; Sadler, 2005).

#### A Solution

The obvious solution for finding hidden disabilities is to look for them. What is not as obvious is how best to do that. One possibility is to help teachers flag potential language problems more consistently and accurately. Teachers are experts at identifying students who struggle to learn and students who frequently misbehave. Helping them to appreciate that language problems may contribute to both is a crucial step. We might advance this message by offering joint SLP and teacher training opportunities at the university level and subsequent joint SLP and teacher professional development opportunities postgraduation. Also, having SLPs work in collaboration with teachers in the classroom might be useful in enhancing teachers' awareness of DLD and its potential to impact learning and behavior.

We must also be able to demonstrate that the language interventions we provide after identification are valuable. Teachers and families may be reticent to begin the identification and assessment process if they perceive the benefits to be minimal. Research demonstrating that language intervention improves not only spoken language but also academic outcomes is critical. These could include knowledge outcomes in subjects such as social studies or science (see Curran & Van Horne, 2019, for a preliminary study of science outcomes among children with DLD who received recast intervention) and skill outcomes in reading, spelling, writing, and calculation (see Tomblin et al., 2020, for the effect of intervention on skill outcomes in children with hearing loss as an example).

An additional step is to develop and employ tools to aid identification. Language screenings are one such tool. Screening programs of various sorts are frequent in U.S. schools, but their content varies from state to state. Many conduct health screenings to detect potential problems with vision, hearing, asthma, scoliosis, blood pressure, dental health, and mental health (Gracy et al., 2018). Screenings of academic areas are also ubiquitous. The U.S. Department of Education (2017) endorses school-wide reading screening as a critical step in identifying children at risk for reading and learning problems.

The massive variability in language development between and within individual toddlers and young preschoolers bodes against successful language screening at early ages (Siu, 2015), but upon kindergarten entry, profiles are more stable, and screenings at or after that point are more likely to be efficacious. To be feasible in a busy school setting, a language screening tool should enable quick administration and scoring without sacrificing the reliability of the results.

To be useful, the tool should have excellent sensitivity for detecting potential cases and specificity for ruling out noncases; it must be robust to cultural and linguistic variations among test takers. We also need evidence of the predictive validity of screening tools. In other words, we need to know whether the children who fail the screener are those who are likely to struggle with communication, learning, and academic outcomes in the years ahead. Without this information, we risk a high rate of false positives and unnecessary alarm among families.

Currently, we lack language screening tools that are known to satisfy all desired criteria. A promising approach is to design screeners that tap known clinical markers of DLD. One such marker is poor sentence recall. In a sentence recall (imitation/repetition) task, the child hears a series of spoken sentences and is asked to repeat each one. Successful repetition reflects short-term verbal memory as well as vocabulary knowledge and grammatical skills (Archibald & Joanisse, 2009). People with DLD are typically poorer at repeating sentences than their age-mates; in fact, the problem is so characteristic that it is considered a clinical marker (Conti-Ramsden et al., 2001). Can we apply the sentence recall task as a quick, valid, and reliable screener for identifying potential cases of DLD? The evidence to date is supportive.

Archibald and Joanisse (2009) administered the Redmond (2005) sentence recall task to 400 children in Grades K–3. Each of the child's 16 sentence repetitions is scored as 0 if there are four or more errors, 1 if there are two or three errors, and 2 if there are no errors. Administration and scoring require about 5 min per child. Eighty-eight children from the larger group of 400 participated in more extensive testing that included administration of the Clinical Evaluation of Language Fundamentals–Fourth Edition (CELF-4; Semel et al., 2003) so that potential cases and noncases could be identified. When a standard score lower than 86 on the CELF-4 was used to identify cases and performance below the 10th percentile on the screener was considered the pass/fail cutoff, the sensitivity of the sentence recall task was .846, and the specificity was .903.

Redmond et al. (2019) administered the Redmond (2005) sentence recall task to 1,060 students in Grades K–3; 254 participated in more extensive follow-up testing to determine cases of DLD. When a standard score lower than 80 on the CELF-4 was used to identify cases, the sensitivity of the sentence recall task was .878, and the specificity was .887 (Redmond et al., 2019).

Plante and Vance (1995) judge screening tests to be good if their sensitivity is at least 90% and their specificity is at least 80%. Thus, in the evaluations of the Redmond (2005) sentence recall task conducted independently by Archibald and Joanisse (2009) and Redmond et al. (2019) with two different large samples, specificity was good, but sensitivity was lower than desired. That does not mean that the task is not useful. Because of its specificity, it is unlikely to overidentify many children; if it is used in conjunction with observation or other measures, concerns about sensitivity leading to underidentification may be reduced.

Poor sentence recall is not the only clinical marker of DLD that could serve as a focus of language screening. Other examples include nonword repetition deficits (see the screening procedure in Seymour et al., 2003) and problems with grammatical sentence comprehension (see procedures in Hendricks et al., 2019). Language screenings could go far toward reducing the inequities in identification if the screening tools are robust in the face of gender, ethnic, and linguistic variation. Researchers should set further development of valid, quick, and equitable language screenings as a high priority.

I encourage school SLPs to view language screenings as a complement to, rather than a replacement for, RTI frameworks that characterize eligibility identification in many U.S. schools. Screenings may enable earlier or more accurate identification of those most at risk of slow progress, by supplementing the most commonly used measures of progress—reading measures—with information about spoken language abilities (Adlof & Hogan, 2019). Children can languish in Tier 1 of RTI for many reasons; a language screener might help when determining whether spoken language should be evaluated as a potential reason. Ultimately, screeners that are valid and efficient may become a useful means by which we can accomplish the Child Find mandate required by U.S. law (IDEA, 2004). In countries where language services are provided outside of an educational context, screenings that teachers can conduct could provide a basis for referral to those services.

### Reason 3: Outdated Policies Constrain Equitable Access to Services

Some barriers to identification reflect entrenched policy decisions in institutions of government, education, and health care. For example, insurance coverage for children with developmental speech-language impairments in the United States is far from common. Some insurers deny coverage for children with DLD because the problem is considered a delay. Others deny coverage because the services are considered educational, in other words, because the child can receive service at school (ASHA, n.d.).

Viewing DLD as a delay that a child will eventually grow out of is wrong. Although some children do catch up to peers during their preschool years, those who present with DLD upon school entry are likely to manifest symptoms as adults (Tomblin et al., 2003). Like ADHD and ASD, DLD can be a lifelong disorder. Moreover, viewing DLD or any other developmental disorders of learning and communication as solely an educational problem does not reflect advances in our understanding of the neural basis of these disorders (e.g., Neul, 2011; Schumann et al., 2011). In 2017, ASHA published a brochure to make a case for the need, benefits, and cost-effectiveness of insurance coverage for developmental speech, language, and hearing disorders. It states explicitly that “developmental delays are the result not the cause of developmental disorders which are rightly considered to be medical conditions.” Simply put, these two reasons for denying coverage of DLD—because it is a delay that requires educational interventions alone—are invalid.

The denials are also inequitable. Other neurodevelopmental conditions are more likely to be covered; ASD is one example. In contrast to DLD, ASD is diagnosed in a medical setting, and in many cases, interventions are, in part, pharmaceutical. ASD is more widely recognized, and the parents and family members of children with ASD are actively engaged in advocacy and political lobbying efforts. These factors likely contribute to differences in coverage. Prior to the turn of the millennium, coverage for ASD was rare, and as is now the case for DLD, one of the often-cited reasons was that the needed treatments were “educational” and not “medical” (Unumb, 2015). However, in the United States, fully funded insurance plans are subject to mandates from state legislatures. Politically active families have been able to convince their legislatures that ASD is a medical condition. They have effectively made the case that providing coverage so that people with ASD can reach their potential is not only socially but also fiscally responsible (Unumb, 2015). As of 2017, 46 states and the District of Columbia had mandated coverage for the diagnosis and long-term treatment of ASD (National Conference of State Legislatures, 2018).

#### A Solution

In the United States, families of children with DLD are not yet prepared for the political advocacy efforts required to instigate change. They cannot find others who share their experience if there is no clear, consistent diagnosis communicated to them; they cannot make the arguments needed if they have a limited understanding of the disorder. It is our responsibility to help prepare them by giving them access to accurate information. When policies are based on myths, misinformation, or outdated evidence, we must educate. Researchers and clinicians in the field must ensure that evidence-based information is accessible to families and policy makers. Undoubtedly, we will need the power of our national organization behind us.

Law et al. (2013) encourage SLPs to take a seat at the policy table. We must promote understanding of spoken language development as well as its facilitation and importance to learning and health. In the long run, the betterment of health and education policies is an act of prevention. Such actions can, ultimately, serve to lower the incidence of clinically significant language challenges in a world that increasingly demands high levels of language competence from its citizens.

### Reason 4: Educational Culture Constrains the Diagnosis of DLD

The vast majority of children who qualify for educational services in the categories of autism, hearing impairment and deafness, visual impairment, traumatic brain injury, orthopedic impairment, and emotional disturbance benefit from a two-armed approach to care, that is, one medical and the other educational. For example, it is likely that a medical audiologist originally diagnoses hearing impairment, and an educational team subsequently determines the disability. Likewise, an autism team in a clinical or medical setting will diagnose ASD, whereas an educational team, again, will determine disability in the school context. In contrast, in the United States, very few children with DLD are diagnosed outside of the educational setting. The medical/clinical arm is typically not available.

The medical model is mostly rejected in U.S. public schools, in large part because of the right-minded philosophy that supports should be provided based on need and not on a diagnostic category. That said, the first step toward assessing needs is the identification or, in medical terms, diagnosis of an impairment. When undertaking that step, the SLP is being asked to apply a medical model in an educational environment. Moreover, the SLP in a school setting may not have the opportunity to take a team approach to diagnosis, making it impossible to rule out other diagnoses that may be more accurate. Even when the school SLP is confident in the diagnosis of DLD, she may find that the mismatch between her role as a “pathologist” and the culture of the educational setting inhibits her from sharing this diagnosis with parents. This dissonance has unfortunate consequences for families living with DLD.

Anecdotally, I have heard from numerous school SLPs who say they are told not to diagnose or not to label the diagnosis (and plenty who say they encounter no barriers to diagnosis). Recent research suggests that barriers do exist. In extensive interviews with mothers of children who have DLD, it is clear that SLPs in school settings often give parents vague labels (e.g., language problem), irrelevant labels (e.g., speech problem), or no diagnostic label at all (Ash et al., 2020). Not surprisingly, the parents are confused. The lack of diagnosis interferes with their understanding of the nature of DLD, their child's prognosis, and their role in helping their child. Parents report that they want a diagnostic label. They feel better able to understand and to explain childhood language disorder to others if they have both a description and a label (Betz & Steigerwald, 2018).

#### A Solution

One solution is to do a bit more of myth-busting. There is nothing in federal law that prohibits an SLP from sharing diagnostic information with a family. A parallel issue arose around the use of the term *dyslexia* in U.S. public schools. In response, the Assistant Secretary of the U.S. Department of Education issued a Dear Colleague letter to clarify that IDEA does not prohibit the use of such terms. He goes on to note that there are situations where the child's parents and the professionals who work with the child would find it helpful to have access to information about the child's specific diagnosis (U.S. Department of Education, 2015). If district policies constrain that information sharing, we must arm SLPs with the evidence they need to challenge those policies.

An additional solution is to create interprofessional models of service delivery for children with DLD. For example, Liu et al. (2018) propose an interprofessional team approach to the differential diagnosis of DLD. They recommend a core team composed of an SLP, a pediatric psychologist, and a developmental pediatrician, with other professionals such as an audiologist, an occupational therapist, a social worker, or a special education teacher, added according to the needs of the child. A differential diagnosis derived in this way would not replace the work done in schools but would complement it by allowing the diagnosis of impairment to be separate from the determination of its educational impact. Such models could also enable receipt of interventions that complement those provided at school or interventions that stand alone when the child is not eligible for services at school. Of course, changes in insurance coverage are required to ensure that this model is equitable for all.

## A Call to Action

Children with DLD are not receiving the attention they deserve from our profession. Laypeople are unaware of the importance of spoken language development. Parents of children who have DLD do not have the words to label it, talk about it, or understand it. For decades, we have known that too many children are going unidentified and that some children are particularly likely to be missed. For decades, we have known that too little research is devoted to DLD. These problems—lack of awareness, lack of service, and lack of research—feed each other. A paradigm shift is needed.

In the United States, school SLPs will always be essential to the appropriate management of DLD. At the same time, the onus is not on school SLPs alone. Those of us who do not work in schools—medical and community SLPs, researchers, and policy makers—must support the continued efforts of school SLPs to provide excellent service to children with DLD. This work will require advocacy from us in collaboration with our national organization, clear communication with the families we serve, the development of new tools, and the formation of new partnerships with schools. Also, as a field, we must develop and implement models for prevention and service delivery that take place outside of the school setting and that complement the work of school-based professionals. No one action will remedy our failures to support children with DLD. I hope that our combined actions will enable innovative solutions to long-standing problems that hamper service, knowledge generation, and advocacy for these children and their families.

## Supplementary Material

10.1044/2020_LSHSS-20-00003SMS1 Supplemental Material S1 Search terms.
