# Supplementary material for: How We Fail Children With Developmental Language Disorder
Source: Lang Speech Hear Serv Sch. 2020 Aug 5;51(4):981–92. doi: 10.1044/2020_LSHSS-20-00003 (PMC7842848; doi:10.1044/2020_LSHSS-20-00003)
Supplement: Supplemental Material S1 [file LSHSS-51-981-s001.pdf]

Supplemental Material S1. Search terms.

**Disorder**	**2000-2009**	**2010-2019**	**Search terms**
Developmental Language Disorder	861	1,388	TI=("specific language impair*" OR ("developmental dysphas*") OR ("developmental aphas*") OR ("developmental language" AND disorder*) OR ("developmental language" AND impair*) OR ("developmental language" AND disab*) OR ("developmental language" AND difficult*) OR (SLI NOT specific impairment potato* gene elegans battery flight arithmetic SLI-1 crystal BMT laser somatostatin gastrin) OR ("language learning disab*"))
Developmental Dyscalculi	81	137	TI=("developmental dyscalcul*" OR ("specific arithmetic* disorder*" NOT dyscalcul*) OR ("math* disorder*" NOT dyscalcul*) OR ("specific arithmetic* retard*" NOT dyscalcul*) OR ("math* retard*" NOT dyscalcul*) OR ("specific arithmetic* disab*" NOT dyscalcul*) OR ("math* disab*" NOT dyscalcul*))
Developmental Dyslexia	2,151	3,047	TI=("developmental dyslex*" OR (dyslex* NOT (acquired OR deep OR surface)) OR ("specific reading disab*") OR ("specific reading retard*") OR ("specific reading impair*") OR ("specific reading difficulties") OR ("developmental reading disorder*"))
Developmental Coordination Disorder	291	764	TI=("developmental coordination disorder*" OR ("developmental co-ordination disorder") OR ("developmental dysprax*") OR ("clumsy child*" NOT dysprax*))
Speech Sound Disorder	280	523	TI=("speech sound disorder" OR ("articulation difficulties" AND child* NOT palate) OR ("articulation disorder*" AND child*) OR ("apraxia of speech" AND (childhood OR developmental)) OR ("delayed speech" OR "speech delay"

			NOT palate) OR ("developmental apraxia of speech") OR ("developmental articulation disab*") OR ("developmental articulation disorder*") OR ("developmental phonological disab*") OR ("developmental phonological disorder*") OR ("developmental phonological impair*") OR ("developmental phonological problem*") OR ("developmental verbal dyspraxia") OR ("phonological disabi*" AND child* NOT developmental) OR ("phonological disorder*" AND child* NOT developmental) OR ("phonological impair*" AND child* NOT developmental) OR ("phonological problems" AND child* NOT developmental) OR ("speech difficulties" AND child*) OR ("speech disorder*" AND child*) OR ("speech impair*" AND child*) OR ("phonological disabi*" AND child* NOT developmental) OR ("phonological disorder*" AND child* NOT developmental) OR ("phonological impair*" AND child* NOT developmental) OR ("phonological problems" AND child* NOT developmental) OR ("speech difficulties" AND child*) OR ("speech disorder*" AND child*) OR ("speech impair*" AND child*))
ADHD	10,686	19,992	TI=("attention deficit hyperactivity disorder*" OR ("attention deficit" NOT (hyperactiv* OR schiz*)) OR (ADHD NOT "attention deficit"))
Tourette Syndrome	952	1,480	TI="Tourette* syndrome"
Intellectual Disability	7,792	11,338	TI=("intellectual disability" OR ("intellectual handicap") OR ("intellectual retardation") OR ("mental handicap") OR ("mental retardation") OR ("learning disab*" NOT specific))
Autism Spectrum Disorder	12,267	38,110	TI=(autism OR (autistic) OR (Asperger OR PDDNOS OR "pervasive developmental disorder" NOT autis*))

Angelman Syndrome	349	543	TI="Angelman syndrome"
Cerebral Palsy	4,367	9,226	TI="Cerebral pals*"
Cornelia de Lange syndrome	220	323	TI=("Cornelia de Lange syndrome" OR "Brachmann-de Lange")
Cri du chat syndrome	60	89	TI=("Cri du chat syndrome" OR "deletion 5p")
Down Syndrome	5,224	7,677	TI=("Down* syndrome" OR "trisomy 21")
Duchenne muscular dystrophy	1,436	3,929	TI="Duchenne muscular dystrophy"
Fetal alcohol syndrome	478<br>576	274<br>748	TI=("fetal alcohol syndrome" OR "foetal alcohol syndrome")<br>TI=("fetal alcohol syndrome" OR "foetal alcohol syndrome" OR "fetal alcohol spectrum disorder" OR "foetal alcohol spectrum disorder")
Fragile X syndrome	937	2,554	TI=("Fragile X syndrome" OR FraX)
Galactosemia	305	336	TI=(Galactosemia OR Galactosaemia)
Kinefelter syndrome	430	805	TI=("Klinefelter* syndrome" OR XXY)
Lesch-Nyhan syndrome	76	73	TI=("Lesch-Nyhan syndrome" OR "Lesch Nyhan syndrome")
Lowe syndrome	72	111	TI=("Lowe syndrome" OR "Lowe's syndrome")
Marfan syndrome	930	1,598	TI="Marfan syndrome"
Neurofibromatosis type 1	1,028	1,994	TI="Neurofibromatosis type 1"
Noonan syndrome	399	687	TI="Noonan syndrome"

PKU	1,276	1,641	TI=(Phenylketonuria OR PKU)
Prader-Willi syndrome	970	1,209	TI="Prader-Willi syndrome"
Rett syndrome	946	1,524	TI=("Rett syndrome" OR "Rett's syndrome")
Rubinstein-Taybi syndrome	116	203	TI="Rubinstein-Taybi syndrome"
Smith-Magenis syndrome	144	133	TI="Smith-Magenis syndrome"
Trisomy 18	251	347	TI=("Trisomy 18" OR "Edwards syndrome")
Tuberous sclerosis	1,432	2,464	TI="Tuberous sclerosis"
Turner syndrome	1,017	1,548	TI=("Turner* syndrome" NOT Ullrich)
Velocardiofacial syndrome	589	1,062	TI=("Velocardiofacial syndrome" OR "Velo-cardio-facial syndrome" OR "22q11.2 deletion")
Williams syndrome	787	1,125	TI=("Williams syndrome" OR "Williams-Beuren syndrome" OR "idiopathic infantile hypercalcaemia" OR "idiopathic infantile hypercalcemia")
XXX	31	35	TI=(XXX AND (female* OR karyotype OR chromosome OR trisomy))
XYY	86	101	TI=XYY

Note: Database settings were All Databases; Advanced search; auto select language to use; auto-suggest publication names ON

The databases included in “all databases” were WOS, BCI, CCC, DRCI, DIIDW, KJD, MEDLINE, RSCI, SCIELO, ZOOREC
